# Hand sanitisers for reducing illness absences in primary school children in New Zealand: a cluster randomised controlled trial study protocol

**DOI:** 10.1186/1745-6215-11-7

**Published:** 2010-01-23

**Authors:** Joanne E McKenzie, Patricia Priest, Rick Audas, Marion R Poore, Cheryl R Brunton, Lesley M Reeves

**Affiliations:** 1 School of Public Health and Preventive Medicine, Monash University, Melbourne, Australia; 2 Department of Preventive and Social Medicine, University of Otago, Dunedin, New Zealand; 3 Department of Economics, University of Otago, Dunedin, New Zealand; 4 Public Health South, Otago District Health Board, Dunedin, New Zealand; 5 Department of Public Health and General Practice, University of Otago, Christchurch, New Zealand

## Abstract

**Background:**

New Zealand has relatively high rates of morbidity and mortality from infectious disease compared with other OECD countries, with infectious disease being more prevalent in children compared with others in the population. Consequences of infectious disease in children may have significant economic and social impact beyond the direct effects of the disease on the health of the child; including absence from school, transmission of infectious disease to other pupils, staff, and family members, and time off work for parents/guardians. Reduction of the transmission of infectious disease between children at schools could be an effective way of reducing the community incidence of infectious disease. Alcohol based no-rinse hand sanitisers provide an alternative hand cleaning technology, for which there is some evidence that they may be effective in achieving this. However, very few studies have investigated the effectiveness of hand sanitisers, and importantly, the potential wider economic implications of this intervention have not been established.

**Aims:**

The primary objective of this trial is to establish if the provision of hand sanitisers in primary schools in the South Island of New Zealand, in addition to an education session on hand hygiene, reduces the incidence rate of absence episodes due to illness in children. In addition, the trial will establish the cost-effectiveness and conduct a cost-benefit analysis of the intervention in this setting.

**Methods/Design:**

A cluster randomised controlled trial will be undertaken to establish the effectiveness and cost-effectiveness of hand sanitisers. Sixty-eight primary schools will be recruited from three regions in the South Island of New Zealand. The schools will be randomised, within region, to receive hand sanitisers and an education session on hand hygiene, or an education session on hand hygiene alone. Fifty pupils from each school in years 1 to 6 (generally aged from 5 to 11 years) will be randomly selected for detailed follow-up about their illness absences, providing a total of 3400 pupils. In addition, absence information will be collected on all children from the school rolls. Investigators not involved in the running of the trial, outcome assessors, and the statistician will be blinded to the group allocation until the analysis is completed.

**Trial registration:**

ACTRN12609000478213

## Background

New Zealand (NZ) has relatively high rates of morbidity and mortality from infectious disease compared with other OECD countries, with infectious diseases, predominantly acute respiratory infections, accounting for 12% of admissions to NZ hospitals [1]. Children, the elderly, and socially disadvantaged groups are known to be at higher risk of infectious disease than others in the population.

One of the important consequences of illness in school aged children is absence from school. Although short absences are unlikely to have any long term effect on children's learning, they are disruptive for the child, their family, and the class. However, infectious illnesses in school children have a number of effects in addition to the direct effects of the disease on the sick child's wellbeing. Transmission from an infected child to other pupils, staff, and family members affects their wellbeing and may require time off school or work for those affected. In addition, when a child needs to stay at home because of illness, caregivers who are in paid work may need to take time off work or pay for care of the child at home. Therefore, childhood infectious diseases may have a significant economic and social impact on the community. However, this has not been well measured.

Reduction of infectious disease requires interruption of person to person transmission. The opportunities for transmission of infectious diseases offered by the school environment are likely to be an important contributor to the rates of infectious illness experienced by children. Given that it is not feasible to require all infectious children to stay away from school (because of asymptomatic transmission, high incidence, and often mild symptoms), reduction of transmission between children at school could be an effective way of reducing the incidence of infectious disease. A 2002 review concluded that "the weight of evidence suggests that personal and environmental hygiene reduces the spread of infection" [2]. There are a number of studies that have assessed the impact of improving hand hygiene to reduce infectious disease incidence in homes [3-6], childcare centres [7,8], University halls of residence [9], and primary schools [10-13]. Meta-analyses have found that handwashing reduces diarrhoea by approximately one third [14,15] and respiratory infection by approximately 20% [14,16].

A recent randomised controlled trial in China [10] found a 42% reduction (p = 0.03) in school absence among children attending schools that were given a handwashing promotion programme and supplied with soap. In developed countries, soap, water, and drying facilities are frequently provided in schools; compliance with hand washing and drying has not been reported but is likely to be poor based on observational studies of older students [17].

Alcohol-based no-rinse hand sanitiser is an alternative hand cleaning technology, which requires only the liquid and a dispenser, no drying facilities, and needs minimum maintenance (refilling the dispenser as required). A recent meta-analysis of hand hygiene interventions in a range of community settings found that hand sanitisers reduced the rate of gastrointestinal illness and of 'combined illnesses' (e.g. school absences), but that there were very few studies and further, definitive studies are still required [14]. Importantly, no information was provided on the potential cost-effectiveness of the intervention.

### Trial objectives

The primary objective of the trial is to establish if the provision of hand sanitisers in primary schools in the South Island of NZ, in addition to an education session on hand hygiene, reduces the incidence rate of absence episodes due to illness in children. Secondary objectives include whether hand sanitisers are effective in reducing the (i) incidence rate of respiratory illness absence episodes, (ii) incidence rate of gastrointestinal illness absence episodes, (iii) average length of illness episode, (iv) incidence rate of absence for any reason, (iv) average length of absence episode, (v) average number of household members who become ill within one week of the participating child's illness onset. We will examine if the intervention is associated with any skin reactions. We will also undertake a cost-effectiveness analysis, providing incremental cost-effectiveness ratios for the cost per absence episode due to illness avoided, the cost per absence attributable to illness, and the cost per days of work lost for parents/guardians (hereafter referred to as parents). We will also conduct a cost-benefit analysis, with the benefits being measured using parents' reported willingness-to-pay (WTP).

## Methods/Design

### Trial design

The design of the trial will be a cluster randomised controlled trial (C-RCT). While this design is less efficient compared to an individually RCT, and will therefore require more children, it has been chosen for several reasons. This design will reduce contamination which would be likely to occur if students within the same school were allocated to both the hand sanitiser and standard hand washing interventions. Second, this design evaluates the effectiveness of how the intervention would be implemented in practice; with hand sanitisers installed in schools. The effectiveness of the intervention may vary if only some children within schools used the hand sanitiser compared to all children, because of the communicable nature of infectious diseases ('herd-effect'). Finally, it is more feasible to run the trial as a cluster trial since it involves less work for the teachers. As a C-RCT, teachers do not have to monitor which children use the hand sanitisers, as they would in an individually RCT.

**Figure 1:**
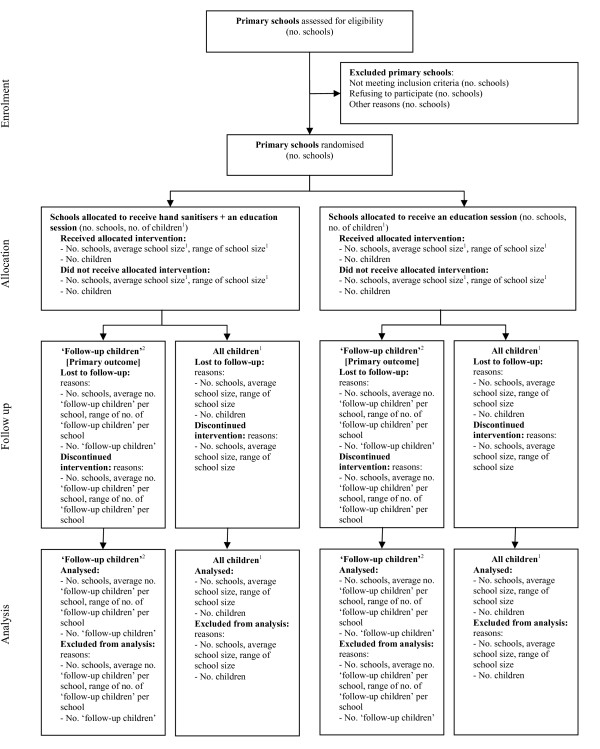
**Flow diagram of the progress of schools and children through the trial**. ^1 ^Includes all children in school years 1 to 6 (generally aged from five to eleven). ^2 ^'Follow-up children' are a randomly selected sample of "All children", whose parents are followed up for detailed information about their illness absences. The primary outcome, absence episodes due to illness, is only measured on this group of children. Figure adapted from Campbell [30].

A random selection of 50 children per school will be selected for whom detailed information about their illness absences will be sought from their parents. These children are referred hereafter as the 'follow-up children'. In addition to the 'follow-up children', absence information will be collected on all children, from the school rolls (Figure 1).

### Eligibility and recruitment

#### Recruitment of schools

A list of all primary schools will be obtained from the NZ Ministry of Education in September 2008. This list will include the total school roll size. Where the school has pupils in years 7 and 8, the school will be contacted to find out the number of pupils in years 1 to 6. Principals of all primary schools in Christchurch City, Dunedin City, and Invercargill City Local Authority Areas, with greater than 100 children in years 1 to 6, will be approached by letter during the last term of 2008, asked to confirm that they currently have at least 100 year 1 to 6 pupils, invited to participate, and asked to respond on a 'fax back' form. Study staff will explain the study procedures further by telephone or in person, as requested. Schools that do not return their fax back form will be contacted by telephone to ask them to do so.

We will follow each school's usual procedures for deciding whether the school will be part of the study. For example, this may involve the principal gaining approval from the Board of Trustees; a group of elected people who are responsible for the governance, control, and management of the school. Based on recent experience from a study of gastrointestinal illness involving NZ primary schools, we estimate that approximately 50% of schools will agree to participate *(A. Ball, personal communication)*.

#### Recruitment of follow-up children

In each school, 50 children will be randomly selected from the school roll in February 2009 by a school liaison research assistant. For each school, a list of numbers between 1 and the total number of children in years 1 to 6 in that school will be randomly sorted. The school liaison research assistant will know what intervention group the school has been assigned to, but will be provided only with the first 50 numbers from the randomly sorted list and a printout of the current school roll by the school administrative staff. They will number the children on the list from 1 to the maximum number, and identify the children whose numbers correspond with the random numbers they have been provided with. They will record the name and class number of those children. The selected children's parents will be contacted by a letter and information sheet sent home via their child, to seek consent for follow-up, should those children be absent from school during terms 2 and 3.

#### Inclusion criteria

Schools will be included if the following criteria are met:

1) There are at least 100 children of primary school age (school years 1 to 6; children will generally range in age from five to eleven years) at November 2008.

2) Schools are currently not using hand sanitiser products or are willing to not use hand sanitiser products for the period of the trial if they are randomised to the control group.

3) Schools are within the city boundaries of Christchurch, Dunedin, or Invercargill in NZ.

4) The principal of the school provides written consent for the school to participate in the trial, be randomised, and potentially receive the hand sanitisers.

Follow-up children will be included if the following criteria are met:

1) Their parents are able to read and understand spoken English to a level where they can read the study information sheet, complete the consent form, and respond to telephone interviews. This will be assessed from a question on the consent form.

2) They attend a year 1 to 6 class in one of the included schools, at the beginning of the second school term in 2009 (the end of April).

#### Exclusion criteria

Schools will be excluded if they are a special needs school.

Follow-up children will be excluded if they meet the following criteria:

1) Their parents are principal investigators or study personnel of the trial.

2) The principal of the primary school directs us not to approach their family.

### Randomisation and allocation concealment

Schools meeting the inclusion criteria will be randomly allocated, within stratum, to receive either the intervention or control. Within each stratum, half the schools will be allocated to the intervention group, and half to the control group. Three strata will be defined by geographical area (Canterbury, Otago, and Southland). Geographical area has been chosen as a stratification variable since we believe this could be strongly predictive of infectious disease if, for example, a flu or gastrointestinal illness affected only selective areas. Consideration was also given to the inclusion of school roll size as a stratification variable since cluster size is fairly commonly used as a stratification variable [18], potentially predictive of absence due to illness, and easily measured. However, an analysis of absence data collected from 12 primary schools in Dunedin over 20 weeks in 2007 revealed no statistically, or clinically, significant association between roll size and absence rate.

Two common methods for implementing restricted randomisation are stratification and matched pairs designs. We have chosen to use stratification in preference to a matched pairs design since the latter design has important limitations. In this C-RCT there are a large number of clusters (68), potentially making it difficult to find matching variables which will form distinct pairs. In addition, if a school opts out of the trial, the entire stratum is removed, resulting in a loss of power [18]. Finally, we wish to be able to calculate estimates of intra-cluster correlation (ICC) for interpretation of the results in this study and for providing estimates for the design of future trials. While this calculation is straightforward when stratification is employed, it is more difficult to estimate when a matched pairs design is used. Certain assumptions, some of which are untestable, have to be assumed for a matched pairs design [19].

The trial statistician (JM) will implement the randomisation. She will only be provided with school codes and district and will randomise the schools to either "A" or "B", so as to remain blinded to the group allocation. Stata/MP 10.1 for Windows [20] will be used to generate the random numbers. PP will randomly allocate "A" and "B" to the intervention and control, prior to receiving the allocation list. Randomisation of all the schools will be undertaken at one time.

### Blinding

It is not possible to blind the children, school administrative staff, or the school liaison research assistants. However, the investigators not involved in the running of the trial (JM, RA, MP, CB), outcome assessors, and statistician (JM) will be blinded until the analysis is completed.

### Interventions

#### Control group

During term 1 2009 (March and April 2009), control schools will have an in-class session, led by the school liaison research assistant, to discuss hand hygiene. The content was developed in collaboration with Public Health Nurses in Dunedin, who have in previous years had a rolling programme of hand hygiene education in schools. The session covers the reasons why and when hand hygiene is important and hand washing technique, including a demonstration of the importance of thorough washing using an ultra-violet light-sensitive cream, Glo Germ™. The same session will be used for the intervention group, with the addition of a discussion about the hand sanitiser. The purpose of this session is to ensure that the two groups are equivalent with respect to hand hygiene knowledge (or at least having had the opportunity to acquire hand hygiene knowledge) at the beginning of the study. The content of the hand hygiene education sessions is available in Additional files 1, 2, 3 (Hand hygiene education session: years 1 and 2; Hand hygiene education session: years 3 and 4; Hand hygiene education session: years 5 and 6).

#### Intervention group

Intervention schools will have the in-class session described above to discuss hand hygiene and to be instructed in the use of the hand sanitiser during term 1, 2009 (March and April 2009). No-touch dispensers will be fitted in all classrooms during the first school holidays in 2009 (April 2009). The hand sanitiser will be at least 60% alcohol.

Children will be able to use the sanitiser at any time they wish, but teachers will be asked to ensure that the children use the hand sanitiser after coughing/sneezing/blowing their nose, and as they leave for morning break and for the lunch break. These times have been chosen for two reasons. First, this will allow the slightly unpleasant smell of the sanitiser (following use by the whole class) to disperse before the children return to the classrooms. Second, break times are when transmission is likely to be higher from children touching one another.

The hand sanitisers will be placed in classrooms to ensure that there will be some teacher oversight of their use, minimising the chances of children playing with the dispensers and sanitiser, and of children getting squirted in the eyes or mouth.

### Timing of recruitment, intervention, and follow-up

School principals will be contacted for potential inclusion in the trial in November 2008. Parents of randomly selected children will be mailed an information sheet and consent form during term 1, March 2009, to seek consent to participate in post-absence interviews.

All schools will be given a single health education session on hand hygiene during term 1, (March and April) 2009. Hand sanitisers will be present in the intervention schools during terms 2 and 3, 27 April to 25 September 2009.

Absence information will be collected for a total of twenty school weeks from the first week of term 2, 27 April 2009. Research assistants will collect absence information from each school weekly. Parents of randomly selected children who have consented to participate in the post-absence interviews will be telephoned eight - nine days after the date of the first day of absence from school.

### Trial outcomes

#### Primary outcome measure

The primary outcome measure for this trial is the number of absence episodes due to *any *illness over 20 weeks; excluding injuries and infestation (head lice and scabies) (Table 1). Absence due to *any *illness has been selected as our primary outcome, in preference to absences due to infectious illness because of difficulties in accurately diagnosing the illness through interviews with the children's parents. The definition of a new episode of absence due to illness has been variously defined between trials. For example, Guinan et al [11] defined a new episode of absence due to illness as one in which there was a lapse of at least five days between a previous period of absence due to illness. Other trials have defined a new episode of absence due to illness as one where the child is absent for at least one day in a given week [10]. We will define a new episode of absence due to illness as one in which there have been at least three days with no absences due to illness. So, for example, if a child is absent from school on Friday, present on Monday, and absent on Tuesday; this will constitute two absences. However, if a child is absent from school on a Tuesday, present on Wednesday, and absent again on Thursday; this will constitute one absence. While there is no theoretical justification for any particular definition for a new episode of absence due to illness, an absence period of at least three days has the advantage of classifying a child who is absent on a Friday and Monday as having only one absence episode, and further, this definition has been used previously [21].

**Table 1:** Outcome measures

Outcome	Collected by	Timing of collection	Source
**Primary outcome**			
Number of absence episodes due to any illness^1,2^	Telephone interview	8 - 9 days after the date of the first day of absence from school	Parents of children
			
**Secondary outcomes**			
*'Follow-up children'*			
Number of absence episodes due to respiratory illness	Telephone interview	8 - 9 days after the date of the first day of absence from school	Parents of children
Number of absence episodes due to gastrointestinal illness	Telephone interview	8 - 9 days after the date of the first day of absence from school	Parents of children
Length of illness episode	Telephone interview	8 - 9 days after the date of the first day of absence from school	Parents of children
Length of absence episode	Telephone interview	8 - 9 days after the date of the first day of absence from school	Parents of children
Number of household members who become ill within one week of the participating child's illness onset	Telephone interview	8 - 9 days after the date of the first day of absence from school	Parents of children
*All children*			
Number of absence episodes for any reason	Liaison research assistant^3^	Weekly	School roll
Length of absence episode	Liaison research assistant^3^	Weekly	School roll
			
**Adverse events**			
Skin reactions^1^	Telephone interview	Following the intervention period	Parents of children

^1 ^'Follow-up children' only.

^2 ^Parents will not be telephoned where it is clear that the child was not unwell (for example, on a holiday, attended a funeral, etc).

^3 ^Through contact with school staff or by extracting the data in person.

#### Secondary outcome measures

Secondary outcome measures collected on the 'follow-up children' will include the number of absence episodes due to respiratory illness, number of absence episodes due to gastrointestinal illness, length of illness episode, length of absence episode, and number of household members who become ill within one week of the participating child's illness onset (Table 1). An illness will be defined as respiratory or gastrointestinal using the definitions applied in a previous trial [13]. A respiratory illness will be defined as an acute illness that includes at least one of the following symptoms: runny nose, stuffy or blocked nose, cough, fever or chills, sore throat, or sneezing. A gastrointestinal illness will be defined as an acute illness that includes at least two watery or much looser than normal bowel movements and stools over a 24 hour period and/or vomiting. The definition of a new episode of absence due to either respiratory or gastrointestinal illness will be defined in the same way as for the primary outcome measure. The length of an absence episode will be defined as the number of days a child was absent from school for a particular episode. The length of the illness episode will be defined as the number of days between the first and last day of absence. For children who are absent on only a Monday or only a Friday, we will define the length of the episode as two days.

Secondary outcome measures collected on all children from the school roll include the number of absence episodes and the length of an absence episode.

#### Adverse events

Following the intervention period, telephone interviews of parents of the 'follow-up children' will be undertaken to collect information on the presence of any skin reactions which may have occurred during the intervention period including: dryness, redness, flakiness, itchiness, eczema, and any other skin reactions.

### Data quality assurance

The follow-up survey of parents will be directly entered into an online program. To enhance the quality of this data, responses to most of the questions will be entered using checkboxes. Absence data collected on all children will be collected weekly by the school liaison research assistants and returned to the study co-ordinator. The study co-ordinator will check this data for invalid codes and dates before adding the data to the database.

### Sample size

The primary outcome in this C-RCT is the number of absence episodes due to illness. In the calculation of sample size for this outcome, adjustment needs to be made for the clustered nature of the design. The variance inflation factor used to achieve this is determined from the average cluster size and the Intra-Cluster Correlation (ICC) [22]. A C-RCT investigating the effectiveness of handwashing promotion in primary school children in China, estimated an ICC of 0.15 for absence prevalence *(A Bowen, personal communication 10 July 2007)*. This estimate has been used in the following calculations. We have powered this trial to detect a 20% reduction in the rate of absence episodes due to illness between arms. If the rate of absence episodes due to illness at 20 weeks follow-up is 2.2 episodes per pupil in the control arm (equivalent to an average of 11 absences per 100 pupil-weeks; the average of 2006/07 winters in Otago/Southland), then a sample of 27 schools per arm, with an average of 50 pupils per school, will be sufficient to detect a 20% reduction in the rate, to 1.76 episodes per pupil per 20 weeks follow-up, with 80% power [23]. This assumes a significance level of 5%.

Allowing for 20% attrition in schools, we plan to initially recruit 34 schools per arm, providing outcome information on a total of 3400 pupils.

### Effectiveness analyses

#### Analysis subsets

The principal of intention-to-treat (ITT) is the appropriate strategy to be used in the primary analysis of the data in this C-RCT for two key reasons. It has the advantage of providing an unbiased estimate of intervention effect because it maintains the balance in prognostic factors between intervention groups, brought about through randomisation. In addition, it allows for non-compliance of hand sanitiser use, therefore providing an estimate of intervention effect which is more reflective of what would occur should the hand sanitisers be implemented in schools [24,25].

Requirements of an ideal ITT analysis include full compliance with the randomised intervention, no missing responses, and follow-up on all participants [24]. In a C-RCT, non-adherence to the intervention and loss to follow-up are more complex than in an individually RCT, since these can occur at multiple levels. In this trial, at the school level, schools in the intervention group may cease using the hand sanitisers while schools in the control group may commence using hand sanitisers, and in addition, schools may withdraw. At the level of the child, there will be non-adherence to the intervention, children may move schools, and parents of 'follow-up children' may withdraw.

We plan to implement procedures to maintain compliance and minimise loss to follow-up. While we expect there will be some attrition, we do not expect this to be large for the primary outcome, or the outcomes collected on all children. For the former, if we are unable to contact the parents' of the 'follow-up children' to ascertain why they were absent, we may be able to determine the reason for the absence from the school administrative staff. For the latter, this data will be collected from the school roll where possible. Therefore, as our primary analysis, we plan to present a modified ITT analysis, where we will analyse participants as they have been randomised, regardless of the intervention they received, but will not impute missing data. As a second approach, we plan to identify potential predictors of missingness through modelling, and include these predictors in the primary analysis model.

A secondary per-protocol analysis will be undertaken where we will only include schools which complied with their allocated intervention. For the intervention group, we will define schools as complying if they used at least 45 ml per child of hand sanitiser solution over the study period. This usage equates to using the hand sanitiser at least once per day.

#### Descriptive analyses at baseline

Descriptive statistics will be presented by intervention group at baseline to compare the comparability of the groups and to provide a description of the population. Summary statistics of demographic and potential confounding variables will be presented.

#### Primary analysis

We will estimate the effectiveness of the hand sanitisers on the number of absence episodes due to any illness with marginal modelling using a generalised estimating equation. This will appropriately account for correlation that will occur between children within the same school. We plan to fit an exchangeable correlation structure, where responses from the same the same school are assumed to be equally correlated [26]. In addition, we will use robust variance estimation which will provide valid standard errors even if the within-school correlation structure has been mis-specified [18]. For this outcome, a negative binomial distribution and link function will be used. This model will include the stratification variable geographical area.

#### Secondary analyses

We will use generalised estimating equations to estimate the effectiveness of the hand sanitisers using appropriate distribution and link functions for the secondary outcomes. As for the primary analysis, we will fit an exchangeable correlation structure and use robust variance estimation. All models will include the stratification variable.

For both the primary and secondary outcomes, we plan to also estimate the effectiveness of hand sanitisers adjusting for the potential confounder, school level deprivation. Research suggests that social economic status may be associated with illness rates [27]. School level deprivation will be measured using the 'decile' assigned to each school by the Ministry of Education for funding purposes. It reflects the proportion of students who live in more deprived communities, using information from the Census on household income, occupation, household crowding, educational qualifications, and income support.

We plan to make no adjustment for multiple testing. All tests will be two-sided and carried out at the 5% level of significance. We will document any changes we make to the study design or statistical analysis plan, or both, in the trial report.

### Economic evaluation

One of the more challenging aspects of this study is determining the economic cost associated with children's illness and subsequent absence from school. Broadly speaking there are two basic approaches we can take to measuring these costs and we will employ both. By capturing individuals' employment circumstances and the amount of time all family members' work time is missed as a result of the illness, we can take a Human Capital approach to estimating the economic impact of the children's illness. While the Human Capital approach is widely used in economic evaluation, its use is particularly limited here as families in which no work time is lost have no cost associated with that illness. As such, we also adopt a WTP approach, asking parents to assign a value to the cost of the illness. Following the techniques described by Liu et al [28], this is done by asking parents to arrive at a figure that they would be prepared to pay to purchase a hypothetical medication that would keep their child from acquiring the same illness (i.e. same symptoms, severity and duration). The main advantage of this approach is that it allows respondents to place whatever values they deem appropriate in terms of inconvenience, lost work time and the pain and suffering of their children.

There is no clear indication in the literature in terms of the best approach to be used to arrive at a WTP figure. To avoid the risk of systematically under or over estimating WTP, each interviewer is randomly assigned an approach for each new respondent. The first approach simply asks the respondent to arrive at a figure on their own. The second approach starts with very low values and works upwards in pre-determined increments. The third approach starts with a very high value and works downwards in pre-determined increments. The questioning sequence used is available in Additional file 4 - Willingness-to-pay questioning sequence.

The analysis will provide a direct comparison between the Human Capital and the WTP approaches. We will also decompose the costs across various sub-populations (i.e. different family structures, ethnic groups, low and high socio-economic status) to determine the extent to which the economic effect of children's illness has a different impact on different types of families, of people with different levels of extended family support and across socio-economic groups (measured by income, education and employment status).

A second objective is to examine the extent to which WTP is affected by the way the question is asked. We believe this will make an important methodological contribution in the collection of WTP data.

### Publication policy

The principal investigator (PP) will ensure that the results from this trial are published regardless of the outcome. Reporting of the trial will adhere to the relevant, and most up-to-date, CONSORT statements at the time of submission [29-31].

### Ethical review

The New Zealand Multi-Region Health and Disability Ethics Committee provided approval for the trial on 13 March 2009 (MEC/09/01/005).

C-RCTs offer additional ethical complexity compared to individually randomised participant trials [32-34], particularly concerning informed consent of individuals. In this trial, the school principals will act as the "gatekeepers"; providing written consent for the school to participate in the trial, be randomised, and potentially receive the hand sanitisers. Parents of all children in the schools allocated to the hand sanitiser group will be contacted with an information sheet explaining the study and informed that hand sanitisers will be fitted in each classroom in their child's school. Parents will be informed that they can opt their child out of using the hand sanitiser at any stage throughout the trial. Parents of the randomly selected sub-sample of children (including children in the intervention and control groups) will be asked to provide written informed consent for the researchers to conduct post-absence interviews with them.

Anonymised data on absences from all children will be collected from the school rolls. Consent for this data collection will not be obtained from parents. Parents of the children in the intervention group will be informed that this data will be colleted, through the information sheet described above. Parents of the children in the control group will not be informed of the anonymised data collection.

## List of abbreviations

C-RCT: Cluster randomised controlled trial; ITT: Intention-to-treat; NZ: New Zealand; OECD: Organisation for Economic Co-operation and Development; WTP: Willingness-to-pay.

## Competing interests

The authors declare that they have no competing interests.

## Authors' contributions

PP, JM, RA, MP and CB conceptualised and designed the trial and secured funding. PP was the lead investigator of the funding application. JM wrote the first draft of this manuscript. PP and RA contributed the background and economic evaluation sections respectively. All authors contributed to revisions of the manuscript have read and approved the final manuscript, and take public responsibility for its content.

## Supplementary Material

Additional file 1 **Hand hygiene education session: years 1 and 2**. This file includes the content of the hand hygiene education session for children in years 1 and 2 (aged approximately 5 and 6 years). In addition, it also details the instructions provided for using the hand sanitisers for children in the intervention group. Click here for file

Additional file 2 **Hand hygiene education session: years 3 and 4**. This file includes the content of the hand hygiene education session for children in years 3 and 4 (aged approximately 7 and 8 years). In addition, it also details the instructions provided for using the hand sanitisers for children in the intervention group. Click here for file

Additional file 3 **Hand hygiene education session: years 5 and 6**. This file includes the content of the hand hygiene education session for children in years 5 and 6 (aged approximately 9 and 10 years). In addition, it also details the instructions provided for using the hand sanitisers for children in the intervention group. Click here for file

Additional file 4 **Willingness-to-pay questioning sequence**. Three questioning approaches to measure a parent's WTP to purchase a hypothetical medication that would keep their child from acquiring the same illness at some point in the future. When interviewers make contact with a parent one of the three approaches to asking the WTP question is randomly assigned. The basic question being asked is whether the approach taking to arrive at a WTP figure is influenced by whether the interviewer starts at a low value and proceeds upwards; starts at a high value and proceeds downwards, or asks the respondent to select a figure without prompting with a starting point. Click here for file
