# Supplementary material for: Hand sanitisers for reducing illness absences in primary school children in New Zealand: a cluster randomised controlled trial study protocol
Source: Trials. 2010 Jan 23;11:7. doi: 10.1186/1745-6215-11-7 (PMC2823737; doi:10.1186/1745-6215-11-7)
Supplement: Additional file 1 — Hand hygiene education session: years 1 and 2. This file includes the content of the hand hygiene education session for children in years 1 and 2 (aged approximately 5 and 6 years). In addition, it also details the instructions provided for using the hand sanitisers for children in the intervention group. [file 1745-6215-11-7-S1.PDF]

# Wash your hands and stay well
# Junior – New Entrant, Year 1 & Year 2

**INTRO**

Name

I'm going to talk to you today about how to wash your hands properly.

**WHY DO WE NEED TO WASH OUR HANDS?**

Get the children to answer.

**READ GERTIE GERM:** A story about hand washing by Eileen Mandell.

**FACTS ABOUT GERMS**

Germs are found all over the world in all kinds of places. They are so small we cannot see them. There are many different types, some are good and some are bad.

Good germs help us turn the food we eat into energy so we can run around and play and they help to keep us healthy.

We even have good and bad germs on our hands, the bad germs on our hands can make us, and others, sick.

**ASK - WHAT ARE SOME OF THE WAYS THAT WE GET GERMS ON OUR HANDS?**

*Get the children to answer then turn the page…*

- When we cough and sneeze in to our hands
- Before you eat food
- If you don't wash your hands after going to the toilet
- Playing in dirty places
- Petting animals

**ASK - WHAT HAPPENS WHEN THE GERMS GET INSIDE US?**

*Get the children to answer then turn the page…*

Germs can make you feel sick like getting a cold or could give you vomiting or diarrhoea. One way they can get inside your body is when you put your hands in your eyes, nose or mouth because remember, you have good and bad germs on your hands all the time.

**ASK - WHAT IS THE BEST WAY TO GET THE GERMS OFF OUR HANDS?**

*Get the children to answer then turn the page…*

By washing your hands properly!

**ASK - SO HOW DO YOU WASH YOUR HANDS PROPERLY?**

*Get the children to answer then turn the page…*

1. Wet hands
2. Add soap
3. Spread soap all over
4. Rub hands together making bubbles at lots of form for 20 seconds (happy birthday song 2x) include your thumb and wrist
5. Rinse well
6. Dry hands for 20 seconds (happy birthday song 2x) on either paper towels, cloth roller or an air dryer, whichever your school uses.

Wash + Dry = Clean Hands

**BUT MY HANDS DON'T LOOK DIRTY**

Remember bugs and germs are on our hands even when they look clean.

**DO Glo Germ™ EXPERIMENT**

- Choose two children, one boy and one girl from the class.
- Put two squirts of Glo Germ™ into both children's hands and then get them to rub it all over their hands.
- Explain to the class that these are 'pretend' germs. Use the torch on each of the children's hands and show the class what the 'pretend' germs look like.
- Ask both children to go and wash their hands.
- While they are doing this ask the class to tell you the 6 steps of washing hands properly again, see if they can remember.
- When the children return shine the UV torch on their hands to see if they have washed the Glo Germ™ off properly then discuss the outcome with the class.

# Intervention Schools – Hand Sanitiser/Dispenser

**What is Hand Sanitiser?**

Hand Sanitiser can be used to kill all the germs on your hands when your hands do not look dirty. When you put the sanitiser on your hands, you rub it all over them like you do with soap, but you don't have to rinse or dry your hands because it dries all by itself.

**What is a dispenser?**

We use a dispenser to put our bottle of hand sanitiser in. The one that will be put in your classroom will have a little light that flashes, this will tell us it is working (show the class). It is automatic so when you put your hands under it, it sprays out some sanitiser and you don't have to touch anything (show the class). Let each child have a turn.

**When will the hand sanitiser be in our classroom, and when will I need to use it?**

The hand sanitiser will be put into your classroom in the $2^{nd}$ and $3^{rd}$ terms of this year. You will need to use it just before you go out to morning tea and just before you go out for your lunch.

**Why are we going to use hand sanitiser?**

We want to see if getting germs off your hands before morning tea and lunchtime stop children getting sick as much.
