# Supplementary material for: Hand sanitisers for reducing illness absences in primary school children in New Zealand: a cluster randomised controlled trial study protocol
Source: Trials. 2010 Jan 23;11:7. doi: 10.1186/1745-6215-11-7 (PMC2823737; doi:10.1186/1745-6215-11-7)
Supplement: Additional file 2 — Hand hygiene education session: years 3 and 4. This file includes the content of the hand hygiene education session for children in years 3 and 4 (aged approximately 7 and 8 years). In addition, it also details the instructions provided for using the hand sanitisers for children in the intervention group. [file 1745-6215-11-7-S2.PDF]

Hand Hygiene

Year 3 & 4 Students

WHAT IS HAND HYGIENE?

HAND WASHING + HAND DRYING = CLEAN HANDS
It was once thought that you only needed to wash hands to make them clean, but now we realise that drying is just as important. This is because moisture left on hands after washing transfers bacteria to other surfaces.

WHY IS HAND HYGIENE IMPORTANT?

It is one of the best and cheapest ways to stop the spread of germs that can cause illnesses.

FACTS ABOUT GERMS

Germs are tiny organisms, or living things, that can cause disease. They are so tiny that you need to use a microscope to see them. Germs are so small and sneaky that they creep into our bodies without being noticed. Germs can be found almost everywhere, they live on almost every surface and even in the air.

There are lots of different types of germs, some good/some bad. Good germs help us turn the food we eat into energy so we can play outside at lunchtime, and they help to keep us healthy. We have good and bad germs on our hands, the bad germs make us, and others, sick.

ASK - WHAT DO YOU KNOW ABOUT GERMS?

Ask - Can we see them when they are on our hands?
No

Ask - Where do they live
Hands, surfaces, animals

Ask - How do they make us sick
In via mouth, nose, eyes

Ask - How do they spread from person to person
Food, surfaces, hands

Ask - How do we prevent them from moving from one person to another?
Washing germs off and drying hands

SO WHEN DO WE NEED TO WASH OUR HANDS?

1) Before we eat
2) If we cough or sneeze into our hands
3) After we have been to the toilet
4) When dirty
5) After petting animals.

WHAT SORT OF SICKNESS COULD WE PREVENT IF WE WASH OUR HANDS AT THESE TIMES?

- Tummy bugs
- Colds/flu
- Sore eyes – conjunctivitis
- Skin infections – school sores

REMIND ME HOW TO WASH MY HANDS PROPERLY?

1. Wet hands
2. Add soap
3. Spread soap all over
4. Rub hands together making bubbles at lots of form for 20 seconds (happy birthday song 2x) include your thumb and wrist
5. Rinse well
6. Dry hands for 20 seconds (happy birthday song 2x) on either paper towels, cloth roller or an air dryer, whichever your school uses.

BUT MY HANDS DON'T LOOK DIRTY

Remember bugs and germs are on our hands even when they look clean.

DO Glo Germ™ EXPERIMENT

- Choose two children, one boy and one girl from the class.
- Put two squirts of Glo Germ™ into both children's hands and then get them to rub it all over their hands.
- Explain to the class that these are 'pretend' germs. Use the torch on each of the children's hands and show the class what the 'pretend' germs look like.
- Ask both children to go and wash their hands.
- While they are doing this ask the class to tell you the 6 steps of washing hands properly again, see if they can remember.
- When the children return shine the UV torch on their hands to see if they have washed the Glo Germ™ off properly then discuss the outcome with the class.

Intervention Schools – Hand Sanitiser/Dispenser

What is Hand Sanitiser?

Hand Sanitiser can be used to kill all the germs on your hands when your hands do not look dirty. When you put the sanitiser on your hands, you rub it all over them like you do with soap, but you don't have to rinse or dry your hands because it dries all by itself.

What is a dispenser?

We use a dispenser to put our bottle of hand sanitiser in. The one that will be put in your classroom will have a little light that flashes, this will tell us it is working (show the class). It is automatic so when you put your hands under it, it sprays out some sanitiser and you don't have to touch anything (show the class). Let each child have a turn.

When will the hand sanitiser be in our classroom, and when will I need to use it?

The hand sanitiser will be put into your classroom in the 2nd and 3rd terms of this year. You will need to use it just before you go out to morning tea and just before you go out for your lunch.

Why are we going to use hand sanitiser?

We want to see if getting germs off your hands before morning tea and lunchtime stop children getting sick as much.
