# Supplementary material for: Hand sanitisers for reducing illness absences in primary school children in New Zealand: a cluster randomised controlled trial study protocol
Source: Trials. 2010 Jan 23;11:7. doi: 10.1186/1745-6215-11-7 (PMC2823737; doi:10.1186/1745-6215-11-7)
Supplement: Additional file 3 — Hand hygiene education session: years 5 and 6. This file includes the content of the hand hygiene education session for children in years 5 and 6 (aged approximately 9 and 10 years). In addition, it also details the instructions provided for using the hand sanitisers for children in the intervention group. [file 1745-6215-11-7-S3.PDF]

Hand Hygiene

Year 5 & 6 Students

WHAT IS HAND HYGIENE?

HAND WASHING + HAND DRYING = CLEAN HANDS

It was once thought that you only needed to wash hands to make them clean, but now we realise that drying is just as important. This is because moisture left on hands after washing transfers bacteria to other surfaces.

WHY IS HAND HYGIENE IMPORTANT?
It is one of the best and cheapest ways to stop the spread of germs that can cause illnesses.

FACTS ABOUT GERMS

Germs are tiny organisms, or living things, that can cause disease. Germs are so small and sneaky that they creep into our bodies without being noticed. They are so tiny that you need to use a microscope to see them. They can be found almost everywhere and live on almost every surface and even in the air.

There are lots of different types of germs, some good/some bad. Good germs help us turn the food we eat into energy so we can play outside at lunchtime, and they help to keep us healthy. We have good germs and bad germs on our hands, the bad germs make us, and others, sick.

HOW DO GERMS SPREAD?

The main way that illnesses like colds and flu spread from one person to the next is when you cough and sneeze. Tiny respiratory droplets, that look like water from a spray bottle, can be inhaled by someone else, or when these droplets fall on to any surface, another person can come along and touch the surface.

Germs can also spread by touching surfaces or objects contaminated with infectious material, such as someone who did not wash their hands after going to the toilet, who then borrows a pencil from you, and gives it back when they have finished. Who, in this classroom, chews on their pencils?

They can spread from person to person via food, surfaces and hands

ASK – THERE ARE TWO MAIN TYPES OF GERMS, BACTERIA AND VIRUSES, CAN YOU NAME ANY OF THEM?

**Viruses names**	**Bacteria names**
Colds (many different types)	Whooping cough (Pertussis)
Flu (influenza viruses)	Bacterial meningitis (often deadly)
Chicken pox	Infections in cuts, stomach etc.
Measles,	
Viral meningitis (not very harmful)	

SO WHEN DO WE NEED TO WASH OUR HANDS?

1) Before we eat
2) After we have been to the toilet
3) When dirty
4) If we cough or sneeze into our hands
5) After petting animals.

WHAT SORT OF SICKNESS COULD WE PREVENT IF WE WASH OUR HANDS AT THESE TIMES?

- Tummy bugs
- Colds/flu
- Sore eyes – conjunctivitis
- Skin infections – school sores

REMIND ME HOW YOU WASH YOUR HANDS PROPERLY?

1. Wet hands
2. Add soap
3. Spread soap all over
4. Rub hands together making bubbles at lots of form for 20 seconds (happy birthday song 2x) include your thumb and wrist
5. Rinse well
6. Dry hands for 20 seconds (happy birthday song 2x) on either paper towels, cloth roller or an air dryer, whichever your school uses.

BUT MY HANDS DON'T LOOK DIRTY

Remember bugs and germs are on our hands even when they look clean.

DO Glo Germ™ EXPERIMENT

- Choose two children, one boy and one girl from the class.
- Put two squirts of Glo Germ™ into both children's hands and then get them to rub it all over their hands.
- Explain to the class that these are 'pretend' germs. Use the torch on each of the children's hands and show the class what the 'pretend' germs look like.
- Ask both children to go and wash their hands.
- While they are doing this ask the class to tell you the 6 steps of washing hands properly again, see if they can remember.
- When the children return shine the UV torch on their hands to see if they have washed the Glo Germ™ off properly then discuss the outcome with the class.

Intervention Schools – Hand Sanitiser/Dispenser

What is Hand Sanitiser?

Hand Sanitiser can be used to kill all the germs on your hands when your hands do not look dirty. When you put the sanitiser on your hands, you rub it all over them like you do with soap, but you don't have to rinse or dry your hands because it dries all by itself.

What is a dispenser?

We use a dispenser to put our bottle of hand sanitiser in. The one that will be put in your classroom will have a little light that flashes, this will tell us it is working (show the class). It is automatic so when you put your hands under it, it sprays out some sanitiser and you don't have to touch anything (show the class). Let each child have a turn.

When will the hand sanitiser be in our classroom, and when will I need to use it?

The hand sanitiser will be put into your classroom in the 2nd and 3rd terms of this year. You will need to use it just before you go out to morning tea and just before you go out for your lunch.

Why are we going to use hand sanitiser?

We want to see if getting germs off your hands before morning tea and lunchtime stop children getting sick as much.
