# Supplementary material for: Hand sanitisers for reducing illness absences in primary school children in New Zealand: a cluster randomised controlled trial study protocol
Source: Trials. 2010 Jan 23;11:7. doi: 10.1186/1745-6215-11-7 (PMC2823737; doi:10.1186/1745-6215-11-7)
Supplement: Additional file 4 — Willingness-to-pay questioning sequence. Three questioning approaches to measure a parent's WTP to purchase a hypothetical medication that would keep their child from acquiring the same illness at some point in the future. When interviewers make contact with a parent one of the three approaches to asking the WTP question is randomly assigned. The basic question being asked is whether the approach taking to arrive at a WTP figure is influenced by whether the interviewer starts at a low value and proceeds upwards; starts at a high value and proceeds downwards, or asks the respondent to select a figure without prompting with a starting point. [file 1745-6215-11-7-S4.PDF]

Willingness-to-pay questioning sequence

When your child gets ill your family incurs losses such as medical costs, lost productivity and missed leisure time. We are now going to ask you a hypothetical question.

Suppose you were told in the next month your child was going to contract another illness, like the one they have just had. Assume you can purchase a special preventative medicine to completely avoid getting this illness again.

Version 1: *How much are you willing to pay for this medicine? Take into account that the money you spend on this will then not be able to be used on other things such as leisure activities. $__________________________*

Version 2: *Think about how much you are willing to pay for this medicine. Take into account that the money you spend on this will then not be able to be used on other things such as leisure activities. Would you be willing to pay $50? $200? $350 $500 (Once they answer no, go down in $50 increments, then $25 increments until you get their maximum willingness-to-pay).*

Version 3: *Think about how much you are willing to pay for this medicine. Take into account that the money you spend on this will then not be able to be used on other things such as leisure activities. Would you be willing to pay $500 $350 $200? $50? (Once they answer yes, go up in $50 increments, then $25 increments until you get their maximum willingness-to-pay).*
